# Case Report: Surgical management and prognostic factors in primary anorectal melanoma: a retrospective analysis of nine cases

**DOI:** 10.3389/fmed.2025.1614614

**Published:** 2025-07-02

**Authors:** Xiangxiang Ren, Xiaoshi Jin, Tianhao Xie, Litao Liu, Qiang Wang, Xingli Sun, Meng Zhang

**Affiliations:** ^1^Department of General Surgery, Affiliated Hospital of Hebei University, Baoding, China; ^2^Department of Dermatology, Affiliated Hospital of Hebei University, Baoding, China

**Keywords:** primary anorectal malignant, melanoma, wide local excision, misdiagnosis, case series

## Abstract

**Background:**

Primary Anorectal malignant melanoma (pARMM) is an exceedingly rare and aggressive malignancy, accounting for approximately 1% of anorectal cancers. It originates from melanocytes in the anorectal mucosa and lacks distinctive clinical features, leading to frequent misdiagnosis and advanced presentation.

**Methods:**

A retrospective analysis was conducted on 9 patients (1 male, 8 females; median age 59 years) with histopathologically and immunohistochemically confirmed ARMM who underwent surgical resection (Wide Local Excision, WLE = 4; Abdominoperineal Resection, APR = 5) and had complete follow-up data (median 19 months, up to May 2025). Diagnostic methods included clinical evaluation, digital rectal exam (DRE), colonoscopy, imaging (CT), histopathology, and immunohistochemistry (IHC). Treatment approaches and outcomes were analyzed.

**Results:**

Common presenting symptoms were hematochezia (44.4%), tenesmus (22.2%), altered bowel habits, anal mass protrusion, or were asymptomatic (11.1% each). DRE revealed exophytic (*n* = 6) or polypoid (*n* = 3) masses. Colonoscopy showed lesions near the dentate line; only 33.3% had obvious pigmentation. IHC positivity: HMB-45/Melan-A 66.7%, S-100 55.6%. Pathological R0 resection was achieved in all patients. During follow-up, 3 patients (33.3%) developed distant metastases (lung, liver), 2 of whom died. Six patients remained disease-free.

**Conclusion:**

Primary Anorectal malignant melanoma (pARMM) often presents with symptoms mimicking common benign anorectal conditions, leading to frequent diagnostic errors. Definitive diagnosis requires histopathological examination, with immunohistochemical markers (HMB-45 and Melan-A positivity) providing critical confirmation. While surgical resection remains the primary treatment, a growing expert consensus supports wide local excision with adequate margins (≥1 cm) as sufficient management. Emerging evidence indicates comparable survival outcomes to more radical procedures in appropriately selected patients.

## Introduction

Malignant melanoma originates from melanocytes predominantly located in the skin. However, melanocytes also reside in mucosal sites, including the gastrointestinal tract, where they may give rise to primary mucosal melanomas (1, 2). Among these, primary anorectal malignant melanoma (pARMM) represents an exceptionally rare entity. pARMM accounts for only 0.4–1.6% of all melanomas and approximately 1% of anorectal malignancies (3–5). It exhibits a striking demographic predilection, predominantly affecting elderly females (male-to-female ratio ≈1:4) (6). Over 50% of patients present with metastatic disease at diagnosis due to delayed detection (7), critically contributing to its poor prognosis (5-year survival <20%) (8). A definitive diagnosis of pARMM necessitates rigorous exclusion of metastatic melanoma from cutaneous, ocular, or other primary sites through comprehensive dermatological examination, imaging, and histopathological correlation (9).

The pathogenesis of pARMM remains incompletely elucidated. Current evidence supports a neural crest origin, wherein precursor melanocytes migrate to the rectal mucosa during embryogenesis (10). Chronic mechanical irritation from fecal transit, coupled with local inflammatory mediators (e.g., COX-2/PGE2 upregulation), is postulated to drive malignant transformation (11). Molecular analyses have identified key driver mutations in: KIT loss-of-function variants (~15–40%), BRAF V600E (~10–25%), and NRAS Q61 mutations (~5–15%) (12, 13). These genetic aberrations constitutively activate the MAPK/ERK signaling pathway, thereby driving melanomagenesis.

pARMM lacks pathognomonic clinical features and frequently mimics benign conditions such as hemorrhoids, leading to a high misdiagnosis rate of 60–70% (12). Common presenting symptoms include hematochezia (60–80%), protrusion of an anal mass (40–60%), and tenesmus or altered bowel habits (20–30%) (14). Notably, 30–40% of cases are amelanotic (15), frequently evading recognition during standard endoscopy. Consequently, immunohistochemistry (IHC) is indispensable for definitive diagnosis, demonstrating positivity for HMB-45/Melan-A (specificity >95%) and S-100 (sensitivity >97% but lower specificity) (16).

Surgical resection remains the cornerstone of management for pARMM; however, the optimal surgical approach is debated. Wide Local Excision (WLE) preserves sphincter function but carries a risk of margin positivity (17), whereas Abdominoperineal Resection (APR) improves local control at the cost of necessitating a permanent colostomy (18). Given the lack of significant survival difference between WLE and APR (HR 1.12, 95% CI 0.87–1.44) shown in recent meta-analyses (19–21), clinical consensus is shifting toward organ-preserving strategies. The role of adjuvant immunotherapy (specifically anti-PD-1/CTLA-4 agents) remains investigative (22).

Due to the rarity of pARMM and persistent controversies surrounding its surgical management, this study aimed to analyze our institutional experience. We conducted a retrospective analysis of nine histologically confirmed pARMM cases treated at our institution to summarize diagnostic and therapeutic experiences, accompanied by a brief literature review. This report aims to provide insights for clinicians managing similar cases.

## Materials and methods

### Patient selection criteria

Patient selection was based on stringent inclusion and exclusion criteria. For inclusion, patients were required to meet *all* of the following conditions: (1) a histopathologically and immunohistochemically confirmed diagnosis of pARMM; (2) availability of complete clinical data, including medical history, physical examination findings, imaging studies, and laboratory results; (3) treatment with surgical resection (either wide local excision [WLE] or abdominoperineal resection [APR]) followed by regular postoperative follow-up; and (4) initial diagnostic workup involving systemic surveillance and multidisciplinary consultations (specifically dermatology and ophthalmology) to definitively exclude cutaneous and extracutaneous primary lesions. Patients were excluded if they met *any* of the following criteria: (1) concurrent diagnosis of another malignancy; (2) confirmed distant metastasis prior to surgical intervention; (3) incomplete clinical data or loss to follow-up; or (4) any prior history of cutaneous or mucosal melanoma.

### Patient characteristics

Nine patients met the inclusion criteria (Table 1), comprising 8 females (88.9%) and 1 male (11.1%). The cohort’s age range was 47–70 years (median 59 years), with symptom duration ranging from 20 to 185 days (median 75 days) before diagnosis. Tumor diameters measured 0.5–3.0 cm (mean 2.4 cm). As mandated by exclusion criterion 4, no patient had a prior melanoma history.

**Table 1:** Patients’ characteristics.

Gender	Age(y)	Disease duration (mo)	Clinical symptoms	Tumor Size (cm)	Lab tests	Diagnosis time	Endoscopic features	Preoperative diagnosis	Surgical approach	Follow-up outcomes
F	67	6	Hematochezia with altered bowel habits	1.5*1.5	N	Outside-hospital diagnosis	Fungating	melanoma	Miles’ operation	24-month recurrence-free
F	70	3	Hematochezia	2.0*2.0	N	Internally diagnosed	Cauliflower-like	hemorrhoids	WLE	12-month recurrence-free
F	67	3	Hematochezia	2.0*3.0	C-199:59 U/mL(0–39)	Internally diagnosed	Cauliflower-like	melanoma	Miles’ operation	18-month recurrence-free
F	53	4	altered bowel habits	2.0*3.0	N	Internally diagnosed	Cauliflower-like	melanoma	Miles’ operation	Peritoneal metastasis at 19 month
F	52	5	Prolapsed anal mass	1.5*2.0	N	Internally diagnosed	Cauliflower-like	hemorrhoids	Miles’ operation	24-month recurrence accompanied by hepatic metastasis
F	69	2	Hematochezia	2.0*2.0	N	Internally diagnosed	Fungating	hemorrhoids	WLE	15-month recurrence-free
F	56	3	Sensation of anal heaviness	2.0*3.0	N	Internally diagnosed	Cauliflower-like	hemorrhoids	Miles’ operation	30-month recurrence-free
M	50	1	Asymptomatic	0.5*0.3	N	Internally diagnosed	Fungating	adenomas	WLE	25-month recurrence accompanied by pulmonary metastasis
F	39	1	Sensation of anal heaviness	3.0*2.0	N	Internally diagnosed	Cauliflower-like	adenomas	WLE	18-month recurrence-free

F, female; M, male; WLE, wide local excision. N, Normal.

### Diagnostic methods

**Clinical presentation was heterogeneous**: hematochezia was the most common symptom (44.4%, *n* = 4), followed by tenesmus (22.2%, *n* = 2), while altered bowel habits, anal mass protrusion, and asymptomatic presentation each occurred in 11.1% (*n* = 1) of cases.

**Digital rectal examination** revealed exophytic, cauliflower-like masses in six patients and polypoid masses in three.

**Figure 1:**
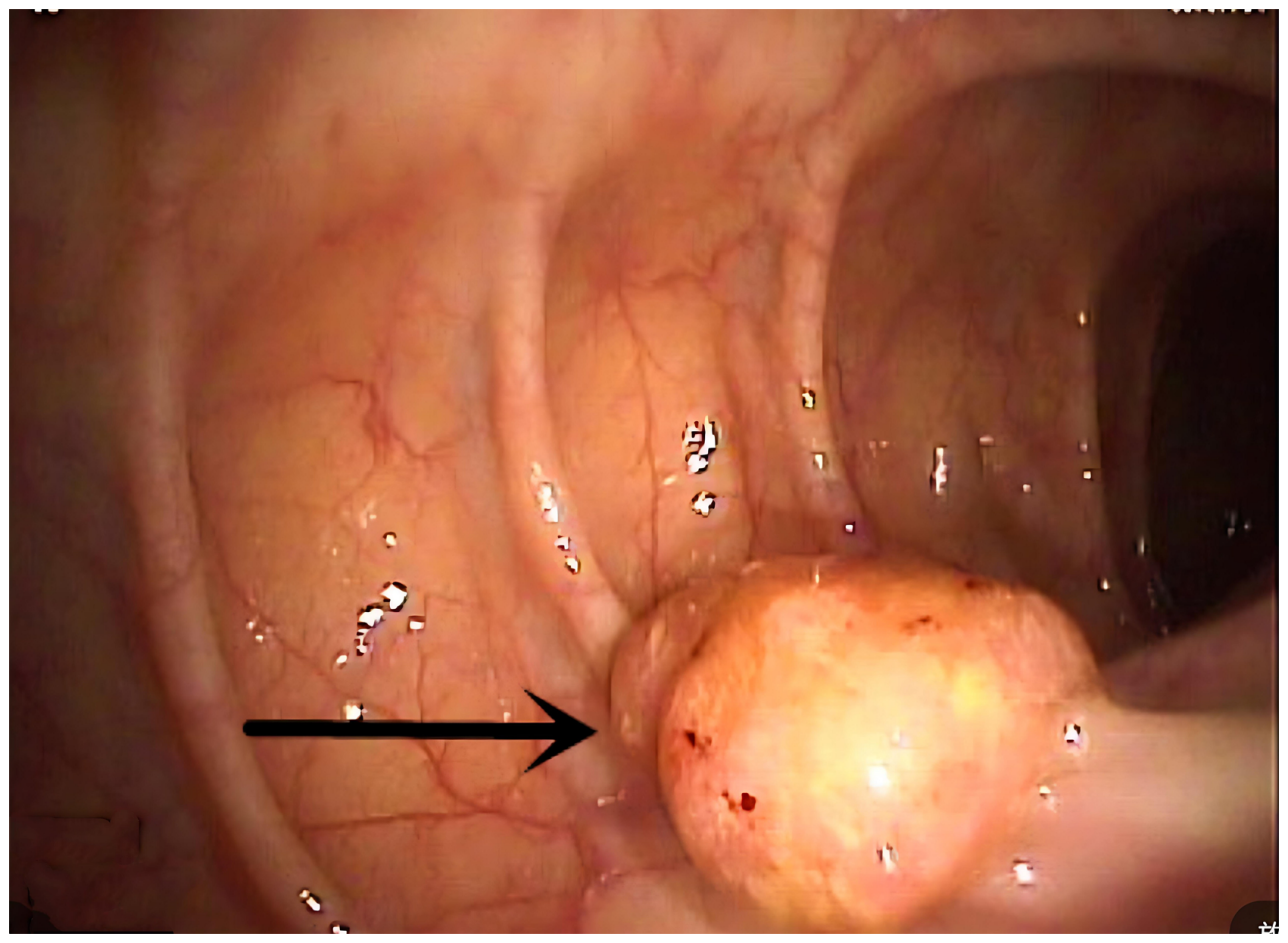
Localized protuberant mass indicated by arrows (arrowheads).

**Endoscopic evaluation** demonstrated that all tumors involved the dentate line region, appearing as sessile protrusions with surface erosion/ulceration, friability, and bleeding tendency (Figure 1). Pigmentation was observed in only three cases (33.3%), absent in the majority (*n* = 6). Esophagogastroduodenoscopy (EGD) was performed only when upper GI symptoms were present, per institutional protocol.

**Contrast-enhanced abdominal CT** uniformly showed focal rectal wall thickening with heterogeneous enhancement, with no distant metastasis detected.

**Cutaneous and Extracutaneous Primary Exclusion** Cutaneous and extracutaneous primary exclusion **were** systematically confirmed through dermatological and ophthalmological evaluations for all patients. No primary cutaneous or ocular melanomas were identified.

**Figure 2:**
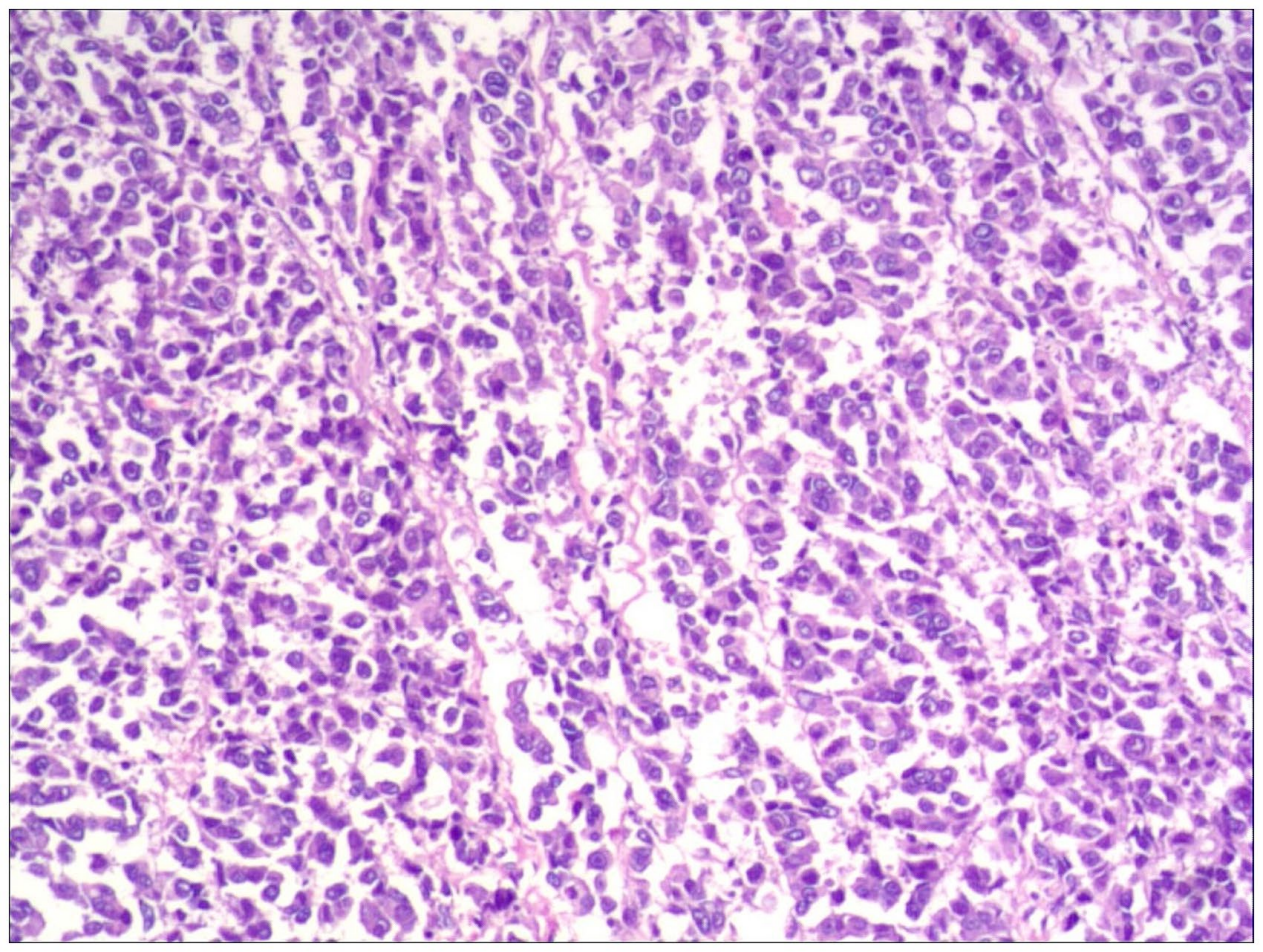
Focal melanin deposition (H&E, ×100).

**Histopathological examination** confirmed R0 resection in all cases, featuring epithelioid or spindled neoplastic cells with nuclear pleomorphism (enlargement, hyperchromasia, prominent nucleoli) and abundant eosinophilic to granular cytoplasm. Tumors exhibited infiltrative growth patterns into smooth muscle and fibrous septa, with associated focal melanin deposition and adjacent mucosal necrosis (Figure 2).

**Immunohistochemistry** demonstrated HMB-45/Melan-A positivity in 66.7% (6/9) and S-100 positivity in 55.6% (5/9) of cases (Table 2)—rates notably lower than typical literature values. Representative immunohistochemical staining patterns were documented: Melan-A positivity showing nested/cord-like brown staining (Supplementary Figure S1), HMB-45 demonstrating patchy/dot-like positivity in a purple cellular background (Supplementary Figure S2), and S-100 exhibiting diffuse/patchy brown staining (Supplementary Figure S3).

**Table 2:** Lymph node metastasis and local tissue invasion and immunohistochemical results in nine cases.

Case	Local invasion	Lymph node metastasis (positive/total)	Vimentin	HBM-45	MelanA	S-100	Ki-67
1	None	1/2	Positive	Positive	Positive	NA	NA
2	NA	NA	Positive	Positive	Positive	Positive	NA
3	Muscular layer	5/6	Positive	Positive	Positive	Negative	70%+
4	Muscular layer	NA	Positive	Focal positive	Negative	Positive	50%+
5	None	None	Positive	Positive	NA	NA	NA
6	NA	NA	Positive	Positive	Positive	Positive	NA
7	Full-thickness	None	Positive	Positive	Positive	Positive	10%+
8	NA	NA	Partial positive	Partial positive	Positive	Positive	NA
9	NA	NA	Positive	Partial positive	NA	NA	NA

NA, Not available (no data recorded) Staining interpretation: Positive (>10% tumor cell staining), Negative (<1% staining), Focal positive (1–10% staining).

Treatment modalities consisted primarily of surgical intervention. Wide local excision (WLE) was performed in four patients, while abdominoperineal resection (APR) was undertaken in five. Surgical approach selection was individualized, integrating tumor characteristics (location relative to the dentate line, diameter, and depth of invasion/T-stage), nodal status (suspicion of metastasis on imaging), and patient preference regarding sphincter preservation. WLE was typically offered for smaller tumors (≤2.5 cm diameter) with superficial invasion (T1/T2) and no evidence of nodal involvement. Conversely, APR was generally indicated for larger tumors (>2.5 cm), deeply invasive lesions (T3/T4), or cases with radiologically suspicious lymph nodes. Adjuvant therapy was administered to only one patient due to high-risk histopathological features (ulceration, high mitotic rate). This patient received a regimen comprising dacarbazine (systemic chemotherapy), temozolomide (for systemic disease control, not prophylactic intent), and sorafenib (initiated based on molecular testing confirming a BRAF V600E mutation).

## Results

### Surgical and pathological outcomes

Surgico-pathological findings are summarized in Table 2. Among the five patients undergoing abdominoperineal resection (APR), four presented with advanced disease: two exhibited lymph node metastasis (pN1), and two demonstrated deep tumor invasion into the perirectal fat (pT3). In the four patients treated with wide local excision (WLE), tumors were confined to the submucosal layer (pT1).

### Survival outcomes

With a median follow-up of 19 months (range: 12–30 months), systemic recurrence occurred in three patients (33.3%), involving the lung (*n* = 1), liver (*n* = 1), and peritoneum (*n* = 1). Disease progression resulted in two deaths. The 12-month and 24-month overall survival rates were 88.9 and 77.8%, respectively. Six patients (66.7%) remained disease-free at the last follow-up assessment.

## Discussion

Our case series of 9 primary anorectal malignant melanoma (pARMM) patients reinforces established characteristics of this rare malignancy while highlighting clinically relevant nuances. The pronounced female predominance (88.9%, 8/9) exceeds the reported male-to-female ratio of ≈1:4 (23), suggesting a more skewed distribution (1:8) than previously documented. Although potentially influenced by limited sample size, this finding warrants investigation into undefined gender-specific biological or environmental factors in pARMM pathogenesis. All cases demonstrated *de novo* primary origins, pathologically confirmed by mucosal melanocytic infiltration and supported by multidisciplinary exclusion of extracutaneous primaries.

A critical diagnostic observation was the high proportion of amelanotic lesions (66.7%, 6/9), exceeding literature estimates (30–40%) (15). This likely contributed to frequent misdiagnosis as benign conditions (e.g., hemorrhoids) and the prolonged median symptom duration (75 days). Notably, IHC marker sensitivity was lower than expected: HMB-45/Melan-A (66.7%) and S-100 (55.6%) fell below reported rates (>95 and >97%, respectively) (16, 24). This discrepancy underscores the need for expanded IHC panels (e.g., *SOX10, MITF*) and expert pathology review, particularly for amelanotic lesions.

Regarding management, our experience supports sphincter-preserving surgery when oncologically appropriate. APR was reserved for larger/deeply invasive tumors (n = 5), while WLE (n = 4) achieved local control in selected superficial cases. Notably, no recurrences occurred in the WLE cohort during follow-up (median 19 months), aligning with meta-analyses showing comparable survival between approaches (19–21). Both disease-specific deaths occurred in the APR group; however, this cohort inherently harbored higher-risk features (nodal metastasis: 2/5; deep invasion: 3/5). This reinforces that tumor biology and staging—rather than surgical extent—drive prognosis, and WLE remains viable for early-stage tumors without compromising intermediate-term outcomes.

Study limitations include its retrospective design, small cohort size (reflecting disease rarity), median follow-up of 19 months (insufficient for long-term survival assessment), and absence of molecular profiling (e.g., *KIT/BRAF/NRAS*), which could elucidate biological heterogeneity.

## Conclusion

In summary, pARMM lacks pathognomonic clinical manifestations, and ancillary diagnostic tools rarely establish a definitive preoperative diagnosis, frequently leading to misdiagnosis or delayed detection. Given its aggressive biological behavior and poor prognosis, early endoscopic biopsy of suspicious anorectal lesions is critical to improve diagnostic accuracy. Surgical resection remains the cornerstone of management, with strategies tailored to individual patient and tumor characteristics and prioritizing quality of life (QoL) preservation. Adjuvant therapies may be considered postoperatively, though evidence supporting survival benefit remains limited. Clinicians must maintain a high index of suspicion for pARMM to minimize diagnostic delays or errors and optimize treatment outcomes.

## Data Availability

The original contributions presented in the study are included in the article/Supplementary material, further inquiries can be directed to the corresponding author.
