# Supplementary figures and images for: Case Report: Surgical management and prognostic factors in primary anorectal melanoma: a retrospective analysis of nine cases
Source: Front Med (Lausanne). 2025 Jul 2;12:1614614. doi: 10.3389/fmed.2025.1614614 (PMC12263572; doi:10.3389/fmed.2025.1614614)

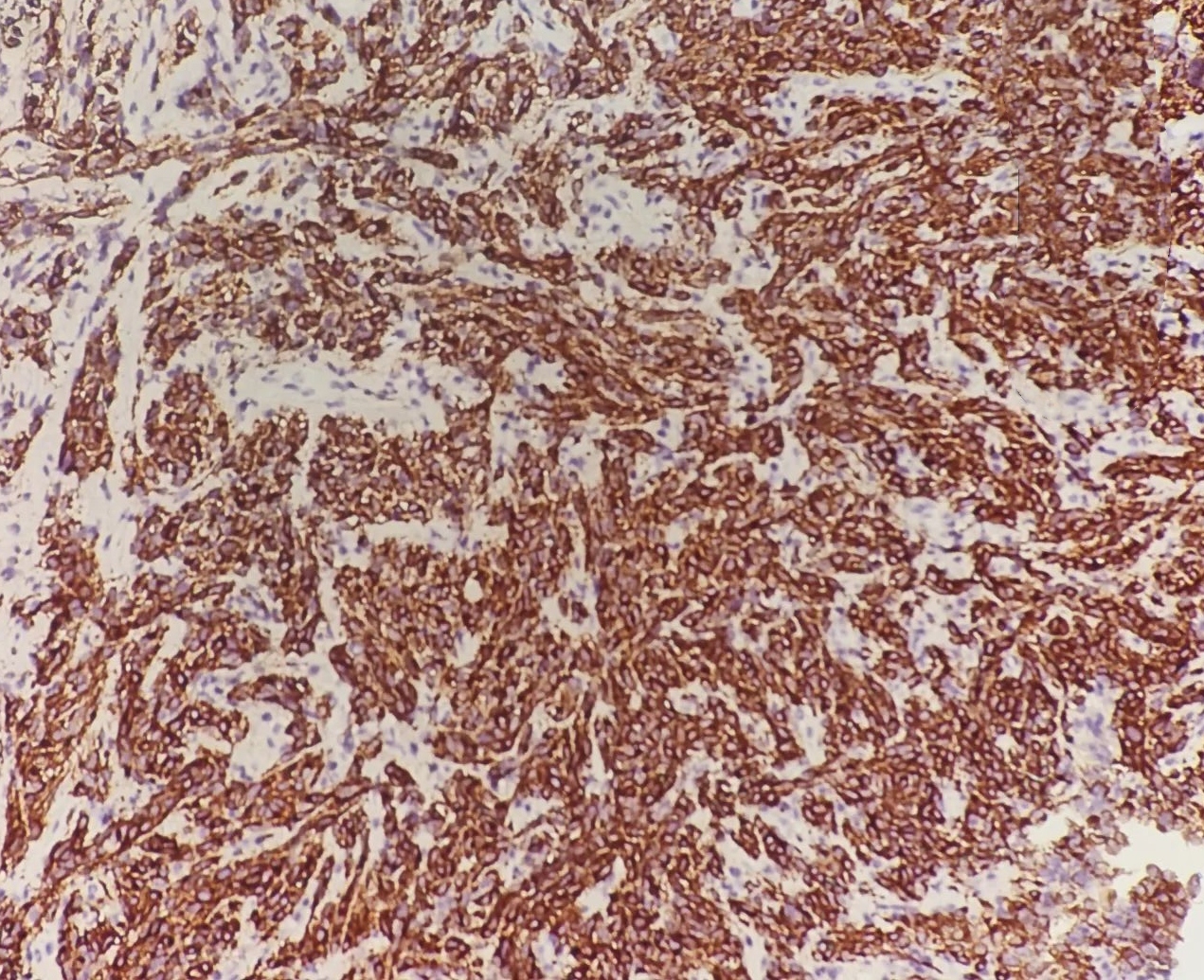

Supplement: Supplementary file 1 [file Image_1.jpeg]

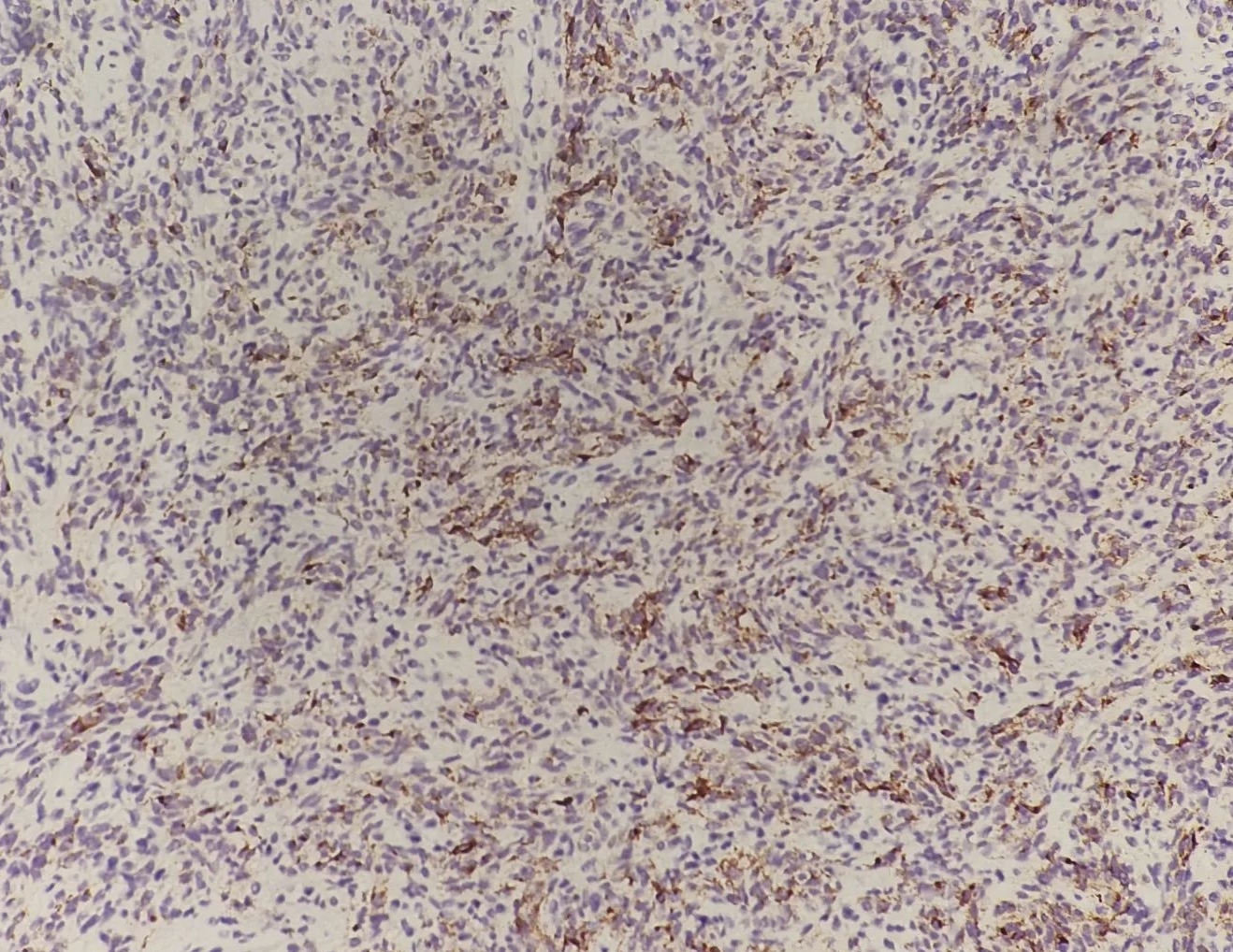

Supplement: Supplementary file 2 [file Image_2.jpeg]

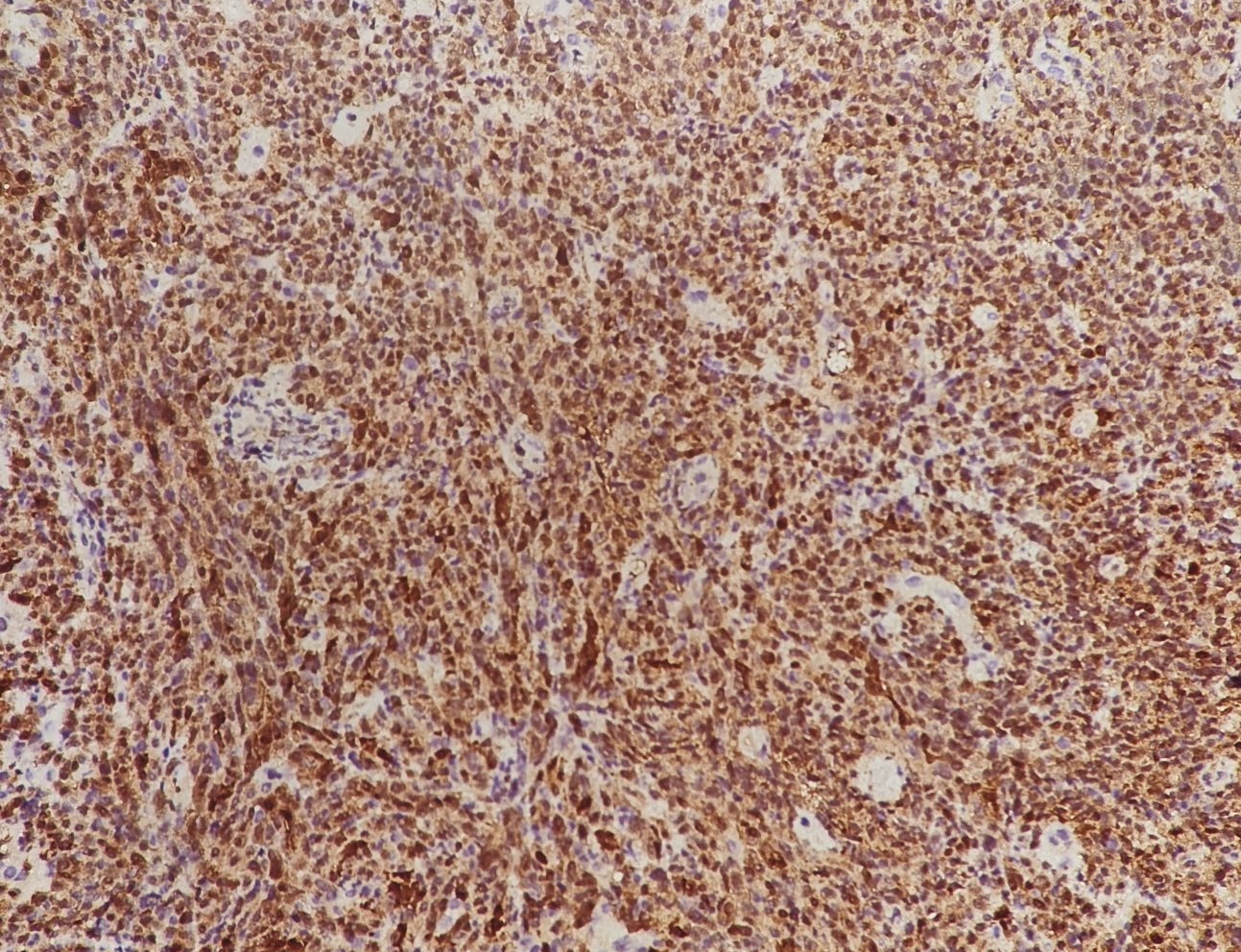

Supplement: Supplementary file 3 [file Image_3.jpeg]
